# Novel prognostic factors and combination therapy outcomes in Morbihan disease: insights from an Asian population

**DOI:** 10.1186/s12886-024-03758-2

**Published:** 2024-11-13

**Authors:** Jungyul Park, Moon-Bum Kim, Hee-young Choi, Suk-woo Yang

**Affiliations:** 1 Department of Ophthalmology, Seoul St. Mary’s Hospital, College of Medicine, The Catholic University of Korea, #222 Banpo-daero, Seocho-gu, Seoul, 06591 Korea; 2 Department of Dermatology, Pusan National University College of Medicine, Busan, Korea; 3 Department of Ophthalmology, Pusan National University College of Medicine, Busan, Korea

**Keywords:** Morbihan disease, Morbihan syndrome, Eyelid lymphedema, Rosaceous lymphedema, Chronic lymphedema

## Abstract

**Purpose:**

This study aimed to identify novel prognostic factors for Morbihan disease (MD) treatment outcomes and evaluate the efficacy of combination therapy in an Asian population, addressing the gaps in current understanding of this rare condition.

**Methods:**

We conducted a retrospective analysis of MD patients diagnosed and treated at a tertiary hospital between 2017 and 2023. Patients received combinations of oral medications (tetracycline, isotretinoin, corticosteroids), topical treatments (tacrolimus, ivermectin), and intralesional steroid injections. Treatment response (TR) was defined as complete symptom remission, while partial remission or recurrence was considered a poor response (PR). Clinical, histological, and biochemical parameters were analyzed to identify prognostic factors.

**Results:**

The study included 24 patients (18 men; mean age 61.3 years). Ten patients (41.7%) achieved TR, while 14 (58.3%) showed PR. Significant prognostic factors for TR included shorter symptom duration (≤ 3 months, *p* = 0.016), lower LDL cholesterol levels (≤ 89 mg/dL, *p* = 0.046), combination treatment with oral and topical medications (*p* = 0.033 at 6 months), and partial response at 1 month (*p* = 0.017). GLMM analysis identified the number of visits (*p* < 0.001), symptom duration (*p* = 0.020), and dyslipidemia (*p* = 0.006) as significant prognostic factors. Histologically, perivascular and perifollicular lymphocytic infiltration were the most common findings (83.3%). Notably, 50% of patients were ANA-positive, challenging previous diagnostic criteria.

**Conclusion:**

This study identifies novel prognostic factors for favorable outcomes in MD, including early intervention and lipid management. Combination therapy, particularly with tacrolimus ointment, shows promise in improving treatment responses. These findings suggest a potential link between lipid metabolism and MD pathophysiology, opening new avenues for targeted therapies.

**Supplementary Information:**

The online version contains supplementary material available at 10.1186/s12886-024-03758-2.

## Introduction

 Morbihan disease (MD), first described by Robert Degos in 1957, is a rare dermatological condition characterized by chronic lymphedema [1, 2]. In the ophthalmic literature, two cases of chronic eyelid lymphedema associated with acne rosacea were first reported in 2000, and the term MD was first mentioned in 2015. More recently, 10 patients with MD were retrospectively reviewed in the UK in 2020 [3–5]. 

The clinical presentation of MD is distinctive, manifesting as chronic erythematous edema predominantly involving the forehead, glabella, eyelids, nose, and cheeks. While these symptoms are often asymptomatic, the chronic nature of the condition can lead to significant facial disfigurement and potential visual field defects, resulting in severe ophthalmological complications if left untreated [3–5]. 

The pathophysiology of MD is complex and multifaceted. Current hypotheses suggest a combination of factors including: (A) Vascular dysfunction: loss of integrity in vascular walls leading to fluid exudation and subsequent swelling. (B) Association with rosacea: MD is often considered an end-stage complication of recurrent vascular dilatation and inflammation associated with rosacea. (C) Lymphatic system impairment: chronic inflammation may lead to damage and remodeling of lymphatic vessels, hindering proper lymphatic drainage and resulting in persistent edema [6–10]. 

Histopathological findings in MD typically include dilated vessels, perifollicular fibrosis, and lymphocytic infiltration around vessels and follicles. Some studies have identified Demodex mites, the primary organism implicated in rosacea, during skin biopsies. However, these histological features are not exclusive to MD, contributing to the ongoing debate about its precise etiology [5, 7, 10–14]. 

The diagnosis of MD presents a significant challenge due to the absence of unique laboratory or histological markers. As a result, MD is often a diagnosis of exclusion, requiring careful differentiation from a wide range of potential conditions that can cause facial edema.

Treatment options for MD remain limited and often yield only partial or transient benefits. Current approaches, based on case reports and small series, include tetracycline-based antibiotics (doxycycline and minocycline), systemic corticosteroids, antihistamines, and isotretinoin have been reported, either alone or in combination. However, the results are often partially or transiently affected, leading clinicians to pursue more invasive treatments, such as local steroid injections and debulking surgery with blepharoplasty or lymphaticovenular anastomosis, often with uncertain outcomes [4, 7–9, 15–19].

Given the rarity of MD and the paucity of large-scale studies, there is a critical need for research that can provide insights into effective management strategies and prognostic factors. Moreover, the majority of existing literature on MD focuses on Western populations, leaving a significant knowledge gap regarding its presentation and management in Asian.

patients.

This study aims to elucidate the clinical and histological features of MD and evaluate the effectiveness of various treatments in an Asian population, specifically focusing on patients of South Korean descent. By identifying novel prognostic factors, we hope to improve the management strategies for MD and provide clearer guidance for clinicians treating this challenging condition.

## Methods

The clinical charts of all the patients diagnosed with MD at a single tertiary national university hospital between January 2017 and January 2023 were retrospectively reviewed. Informed consent was obtained from all the patients. The study protocol was approved by the Institutional Review Board of Pusan National University Hospital (PNUH) and was performed in accordance with the principles of the Declaration of Helsinki. MD was diagnosed based on a comprehensive review of the clinical features and histological findings by an ophthalmologist and a dermatologist. Patients lacking post-treatment records and histological results or those diagnosed histologically after invasive treatments such as debulking surgery or blepharoplasty were excluded.

### Diagnosis and ophthalmological examinations

Patients included in this study met the following criteria: (1) Patients must have no clinical and laboratory signs of idiopathic inflammatory inflammation, IgG4-related orbital disease, thyroid eye disease, angioedema, sarcoidosis, neoplastic associated lymphedema, Melkersson-Rosenthal syndrome or other dermatologic, ophthalmologic conditions causing periorbital lymphedema confirmed by both a dermatologist (MBK) and ophthalmologist (JYP); (2) Patients should have no previous surgical history involving the orbit or eyelid, and no history of trauma, and (3) Diagnosis of MD in both the Dermatology and Ophthalmology departments must be confirmed by clinical, laboratory, and histologic findings. Clinical presentation, duration of symptoms, medical history, laterality, location of lymphedema, laboratory findings, and histopathological findings were assessed before treatment. Eyelid tissues were obtained from office-based punch biopsies and stained with hematoxylin and eosin to identify dermal edema, inflammation, and other structural findings. All histopathological specimens were re-evaluated by a dermatologist with a specialty in dermatopathology (MBK). The extent of lymphocyte infiltration and vascular dilation was assessed by a dermatologist specializing in dermatopathology. These histopathological features were graded as mild, moderate, or severe based on the degree of perivascular or perifollicular lymphocytic infiltration and the extent of telangiectasia. This grading system follows standard dermatopathological practice, for ensuring consistency the evaluation of these features across all specimens.

### Treatment protocol and evaluation

#### Treatment protocol and evaluation

Patients received single or combinations of the following medications: tetracycline (minocycline 100 mg/day), oral corticosteroid (20 mg/day), isotretinoin (10–20 mg/day), tacrolimus ointment (0.1%), ivermectin 1% cream, and intralesional triamcinolone injections (20 mg for each eyelid). Patients were initially evaluated at 1 month after the first treatment to assess symptom improvement and side effects. For those experiencing a poor response or side effects, additional medications were added, or a change in medication was considered. Once a medication regimen was established, its use was continued for at least 4–6 months. Subsequently, evaluations were conducted every 6 months to monitor the response to treatment.

Treatment responses were defined as follows: Treatment Response (TR) involved a subjective report of complete improvement by the patient and noticeable complete improvement of symptoms as assessed by the doctors (JYP and MBK) with no recurrence at follow-up. Poor Response (PR) was defined as a subjective report of partial or no improvement by the patient, with recurrence, partial or no improvement, or progression of symptoms as assessed by doctors.

**Fig. 1:**
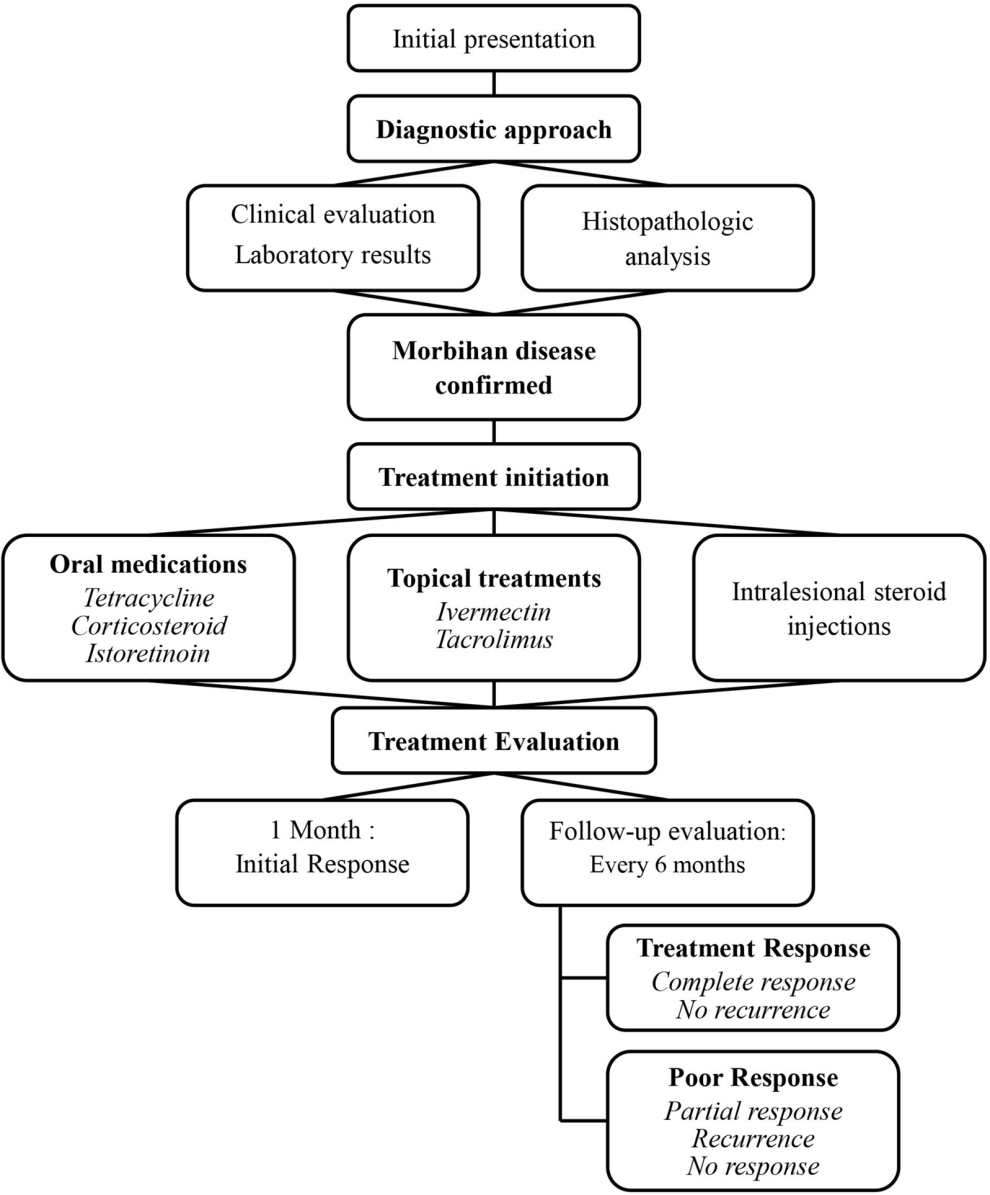
Diagnostic and Treatment Algorithm for Morbihan Disease (MD) This flow chart illustrates the comprehensive approach to diagnosing, managing, and evaluating MD in our study. The process begins with the initial patient presentation and progresses through diagnostic steps, including clinical evaluation, laboratory tests, and histopathological analysis. Once MD is confirmed, the treatment modalities, including oral medications (tetracycline, corticosteroid, isotretinoin), topical treatments (ivermectin, tacrolimus), and intralesional steroid injections were applied in a single or combination manner. The treatment evaluation phase involves an initial response assessment at 1 month, followed by regular follow-ups every 6 months. Treatment outcomes are categorized as either complete response with no recurrence or poor response, which includes partial response, recurrence, or no response

Overall flow chart from initial presentation to evaluation of treatment response are depicted in Fig. 1.

### Statistical analysis

Continuous data were compared using the Student’s t-test or the Mann − Whitney U test. Categorical variables were compared using Fisher’s exact test. Generalized Linear Mixed Model (GLMM) was used to assess the treatment effects over time. The model was fitted using Restricted Maximum Likelihood (REML) to obtain unbiased estimates of variance components. Coefficients (β), 95% confidence intervals (CIs), and p-values were calculated for each fixed effect. The Kolmogorov − Smirnov test was used to assess the normality of data distribution. We identified the cutoff value using the point on the Receiver Operating Characteristic (ROC) curve that had the shortest distance from the upper left corner of the unit square and the highest Youden index. Statistical Package for the Social Sciences (SPSS) software (version 27.0; IBM Corp., Armonk, NY, USA), R (version 4.0.5, available at http://cran.r-project.org), and additional Python 3.8 packages were used to draw Sankey diagrams. Statistical significance was determined using unadjusted two-sided *P*-values of less than 0.05.

## Results

### Patient characteristics and clinical presentation

This study included 24 patients with MD, comprising 18 men (75%) and 6 women (25%), with a mean age of 61.3 ± 2.41 years. The mean duration of symptoms before treatment was 22.0 ± 6.43 months. Ten patients (41.7%) exhibited TR, while 14 (58.3%) had PR.

Eyelid edema was observed in all participants, with unilateral involvement being the most common (17/24, 70.8%). 20% of patients exhibited symptoms on both the upper and lower eyelids simultaneously, and two displayed facial erythematous edema. The TR group had a significantly shorter mean duration of symptoms (14.2 ± 3.72 months) compared to the PR group (27.36 ± 27.02 months, *P* = 0.01) (Table 1).

**Table 1:** Clinical and biochemical characteristics of patients

Baseline Characteristics	Groups			
Variable	Total (*N* = 24)	TR (10, 41.7%)	PR (14, 58.3%)	*P*-value
Men, No. (%)	18 (75)	7 (70%)	11 (78.6)	0.665
Age, mean (SD), y	61.3 (2.41)	63.0 (10.24)	60 (13.02)	0.88
Mean duration of symptoms (months)	22.0 (6.43)	14.2 (37.20)	27.36 (27.02)	0.01^*^
History, No. (%)				
Hypertension	10 (41.7)	4 (40)	6 (42.9)	> 0.99
Diabetes	6 (25)	3 (30)	3 (21.4)	0.665
Dyslipidemia	2 (8.3)	2 (20)	0 (0)	0.163
Stroke	1 (4.2)	1 (10)	0 (0)	0.44
HBV	1 (4.2)	0 (0)	1 (7.1)	> 0.99
Post COVID-19 vaccination	1 (4.2)	0 (0)	1 (7.1)	> 0.99
Clinical presentations, No. (%)				
Eyelid edema	24 (100)	10 (100)	14 (100)	-
Eyelid erythema	23 (95.8)	10 (100)	13 (92.9)	> 0.99
Heat sensation	3 (12.5)	3 (30)	0 (0)	0.059
Itching	2 (8.3)	0 (0)	2 (14.3)	> 0.99
Papules	2 (8.3)	2 (20)	0 (0)	0.163
Pain	0 (0)	-	-	-
Laterality, No. (%)				0.151
Right	7 (29.2)	5 (50)	2 (14.3)	
Left	10 (41.6)	2 (20)	7 (50)	
Both	7 (29.2)	3 (30)	5 (35.7)	
Location, No. (%)				0.687
Upper eyelid	15 (62.5)	5 (50)	8 (57.1)	
Lower eyelid	2 (8.3)	0 (0)	2 (14.3)	
Upper and lower eyelid	5 (20.8)	3 (30)	3 (21.4)	
Facial	2 (8.3)	2 (20)	1 (7.1)	
Laboratory abnormalities				
ANA+, No./Total No. (%)	10/20 (50)	6/10 (60)	4/10 (40)	> 0.99
Patterns of ANA, No. (%)				> 0.99
Speckled	4 (40)	3 (50)	1 (7.1)	
Homogenous	4 (40)	2 (33.3)	2 (14.3)	
Nucleolar	2 (20)	1 (16.7)	1 (7.1)	
Dyslipidemia, No./Total No. (%)	11/18 (61.1)	2/7 (28.6)	9/10 (90)	0.013^*^
HDL-C (< 40 mg/dL)	5/18 (27.8)	1/8 (12.5)	5/10 (50)	0.023^*^
LDL-C (> 100 mg/dL)	9/15 (60)	3/8 (37.5)	7/8 (87.5)	0.041^*^
Cholesterol (> 200 mg/dL)	8/18 (44.4)	1/8 (12.5)	5/10 (50)	0.664
Triglyceride (> 200 mg/dL)	7/18 (38.9)	2/9 (22.2)	6/10 (60)	0.066
HDL-C (mg/dL)	52.22 (12.45)	55.13 (6.60)	49.9 (15.70)	0.18
LDL-C (mg/dL)	113.79 (39.94)	93.57 (13.54)	131.49 (36.39)	0.033^*^
Cholesterol (mg/dL)	189.67 (41.12)	167.75 (38.80)	207.2 (35.41)	0.037^*^
Triglyceride (mg/dL)	167.61 (80.46)	119.13 (43.98)	206.4 (83.43)	0.021^*^
ESR (> 12 mm/h), No./Total No. (%)	6/16 (37.5)	2/7 (28.6)	4/9 (44.4)	0.633
ANCA	0 (0)	-	-	-
ACE	0 (0)	-	-	-
RF	0 (0)	-	-	-

*TR* treatment response; *PR* poor response; *y* years; *Mo* months; *No* number; *SD* standard deviation; *HBV* hepatitis B virus; *ANA* anti-neutrophil antibodies; *HDL-C* high-density lipoprotein cholesterol; *LDL-C* low-density lipoprotein cholesterol; *ESR* erythrocyte sedimentation rate; *ANCA* anti-neutrophil cytoplasmic antibodies; *ACE* angiotensin-converting enzyme; *RF* rheumatoid factor, *COVID-19* coronavirus disease 2019

* *P* < 0.05, considered statistically significant

### Laboratory findings and prognostic factors

Half of the patients tested positive for antinuclear antibodies (ANA), albeit at insignificant levels. Among the 18 patients whose lipid profiles were evaluated, 11 (61.1%) had dyslipidemia. The PR group showed a significantly higher rate of dyslipidemia (9/10, 90%) than the TR group (2/7, 28.6%; *P* = 0.013) (Table 1).

Low-density lipoprotein cholesterol (LDL-C), total cholesterol, and triglyceride levels were all lower in the TR group compared to the PR group. Specifically, LDL-C levels were significantly lower in the TR group (93.57 ± 13.54 mg/dL) compared to the PR group (131.49 ± 36.39 mg/dL, *P* = 0.033). Similarly, total cholesterol levels were lower in the TR group (167.75 ± 38.80 mg/dL) compared to the PR group (207.2 ± 35.41 mg/dL, *P* = 0.037), and triglyceride levels were also lower in the TR group (119.13 ± 43.98 mg/dL) compared to the PR group (206.4 ± 83.43 mg/dL, *P* = 0.021).

However, when categorized into normal and abnormal groups, only LDL-C levels remained statistically significant (*P* = 0.041), while total cholesterol (*P* = 0.664) and triglycerides (*P* = 0.066) did not show a significant difference between the TR and PR groups.

**Fig. 2:**
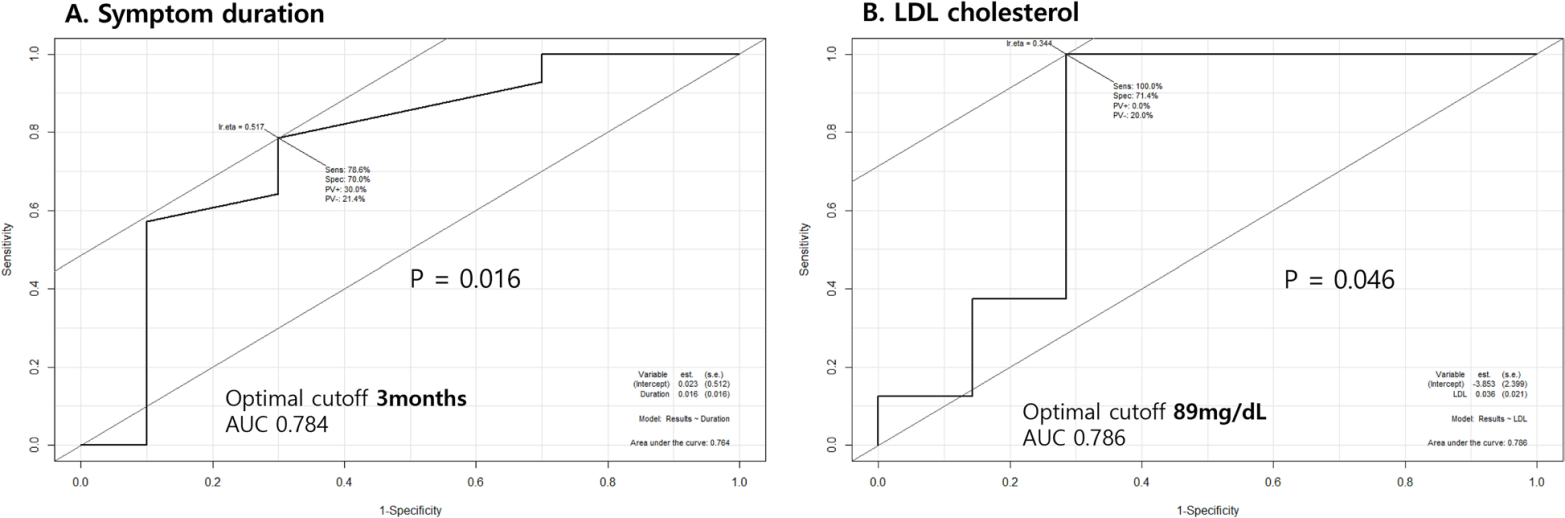
Receiver Operating Characteristic (ROC) Curves for Symptom Duration and Low-density Lipoprotein (LDL) Cholesterol Levels. **A** Symptom Duration: The ROC curve shows an optimal cutoff of 3 months for predicting treatment response, with an area under the curve (AUC) of 0.784 (*P* = 0.016). At this cutoff, the sensitivity is 78.6% and specificity is 70.0%. **B** LDL Cholesterol: The optimal cutoff was identified at 89 mg/dL, with an AUC of 0.786 (*P* = 0.046). This cutoff yields a sensitivity of 100% and specificity of 71.4%. Both parameters demonstrate significant predictive value for treatment response, with symptom duration ≤ 3 months and LDL cholesterol ≤ 89 mg/dL associated with better treatment outcomes

ROC analysis revealed that symptom duration ≤ 3 months (AUC = 0.784, *P* = 0.016) and LDL-C levels ≤ 89 mg/dL (AUC = 0.786, *P* = 0.046) were significant predictors of positive treatment response (Fig. 2).

### Histopathological findings

Perivascular or perifollicular lymphocytic infiltration was the most prevalent finding, present in 83.3% (20/24) of cases, with no significant difference between response groups. Dermal edema was noted in 41.7% (10/24) of patients, and focal granulomatous infiltrations were found in 37.5% (9/24) of patients. Notably, the presence of dilated vessels or telangiectasias was more common in the TR group (50% [5/10]) than in the PR group (14.2% [2/14]), although this difference was not statistically significant (Table 2).

**Table 2:** Histopathologic features of patients

			Group		
	Histopathologic findings	Total (*N* = 24), (%)	Treatment response (*N* = 10), (%)	Poor response (*N* = 14), (%)	*P*-value
1	Perivascular or perifollicular lymphocytic infiltration in the dermis	20 (83.3)	9 (90)	11 (78.6)	> 0.99
2	Dermal edema	10 (41.7)	4 (40)	6 (42.9)	0.697
3	Focal granulomatous infiltrations	9 (37.5)	6 (60)	3 (21.4)	0.206
4	Presence of a few dilated vessels (or telangiectasia)	7 (29.2)	5 (50)	2 (14.2)	0.182
5	Perivascular infiltration of mononuclear cells	3 (12.5)	1 (10)	2 (14.3)	> 0.99
6	Diffuse hemorrhage in the dermis	1 (4.2)	0 (0)	1 (7.1)	> 0.99
7	Demodex in follicle	1 (4.2)	0 (0)	1 (7.1)	> 0.99
8	Degeneration of dermal collagen	1 (4.2)	1 (10)	0 (0)	0.458
9	Reactive epidermal hyperplasia	1 (4.2)	1 (10)	0 (0)	0.458
10	Hyper-melanosis of basal layer	1 (4.2)	1 (10)	0 (0)	0.458

*N* numbers

Statistical significance was set at *P* < 0.05

### Treatment outcomes

The average monitoring time was 12.46 ± 6.31 months, with no significant difference between groups (*P* = 0.977). The first response (complete or partial) to treatment was observed at 3.33 ± 3.94 months, occurring earlier in the TR group (1.90 ± 1.00 months) compared to the PR group (4.63 ± 5.12 months), though this difference was not statistically significant (*P* = 0.282).

At 1 month after treatment, no patient showed TR or complete remission of symptoms. Eleven out of 24 (45.8%) patients showed partial improvement of symptoms (partial response) and 13(54.2%) showed no response to treatment. Partial response at 1 month was significantly associated with complete remission at the final visit (*P* = 0.017), regardless of the specific treatment regimen. No significant differences were observed in relation to the use of specific medications or treatment protocols (*P* > 0.05).

Tetracycline and isotretinoin were the most commonly used oral medications, administered to 70.8% and 66.7% of patients, respectively. Tacrolimus ointment was applied in 79.2% of patients, showing higher effectiveness in the TR group (100% usage) compared to the PR group (64.3%, *P* = 0.053) (Table 3).

**Table 3:** Treatment characteristics of patients

	Groups			
Treatment characteristics	Total (*N* = 24)	Treatment response (*N* = 10)	Poor response (*N* = 14)	*P*-value
Monitoring time, Mo. (SD, min-max)	12.46+-6.31 (6.00 − 30.00)	12.70+-6.53 (6.00 − 24.00)	12.29+-6.39 (6.00 − 30.00)	0.977
First response time, Mo. (SD, min-max)	3.33+-3.94 (1.00 − 18.00)	1.90+-1.00 (1.00 − 3.00)	4.63+-5.12 (1.00 − 18.00)	0.282
Partial response at 1month (%)	11 (45.8)	8 (80)	3 (21.4)	0.017^*^
Complete response time, Mo. (SD, min-max)	-	7.90+-5.72 (2.00 − 17.00)	-	-
Treatment regimen, No. (%)				
Tetracycline P.O	17 (70.8)	8 (80)	9 (64.3)	0.653
Isotretinoin P.O	16 (66.7)	5 (50)	11 (78.6)	0.204
Corticosteroid P.O	11 (45.8)	5 (50)	6 (42.9)	> 0.99
Intralesional steroid inj. (ILS)	14 (58.3)	5 (50)	9 (64.3)	0.678
Tacrolimus ointment	19 (79.2)	10 (100)	9 (64.3)	0.053
Ivermectin ointment	9 (37.5)	4 (40)	5 (35.7)	> 0.99
Others (UDCA, statin, Cipol, metronidazole)	2 (8.3)	0	2 (14.3)	0.493
Duration of medication, Mo. (SD, min-max)				
Tetracycline P.O	3.08+-2.38 (0.00 − 9.00)	3.50+-2.51 (0.00 − 9.00)	2.79 + 2.33 (0.00 − 6.00)	0.666
Isotretinoin P.O	4.00+-3.50 (0.00 − 12.00)	2.70+-2.83 (0.00 − 7.00)	4.93+-3.73 (0.00 − 12.00)	0.212
Corticosteroid P.O	0.75+-1.19 (0.00 − 3.00)	0.70+-1.06 (0.00 − 3.00)	0.79+-1.31 (0.00 − 3.00)	0.886
Tacrolimus ointment	5.79+-4.58 (0.00 − 14.00)	5.80+-3.91 (0.00 − 14.00)	5.79+-5.15 (0.00 − 12.00)	0.977
Ivermectin ointment	2.13+-3.49 (0.00 − 12.00)	2.30+-3.13 (0.00 − 8.00)	2.00+-3.84 (0.00 − 12.00)	0.752
Repetition numbers of ILS, No. (SD, min-max)	1.46+-1.77 (0.00 − 5.00)	1.70+-2.16 (0.00 − 4.00)	1.43+-1.50 (0.00 − 5.00)	0.931
Used drugs, No. (SD, min-max)	3.79+-1.18 (1.00 − 5.00)	3.60+-1.17 (2.00 − 5.00)	3.93+-1.21 (1.00 − 5.00)	0.472

*N* number; *SD* standard deviation; *min,* *max* minimum, maximum; *P.O* per oral; *UDCA* ursodeoxycholic acid

Statistical significance was set at *P* < 0.05

**Fig. 3:**
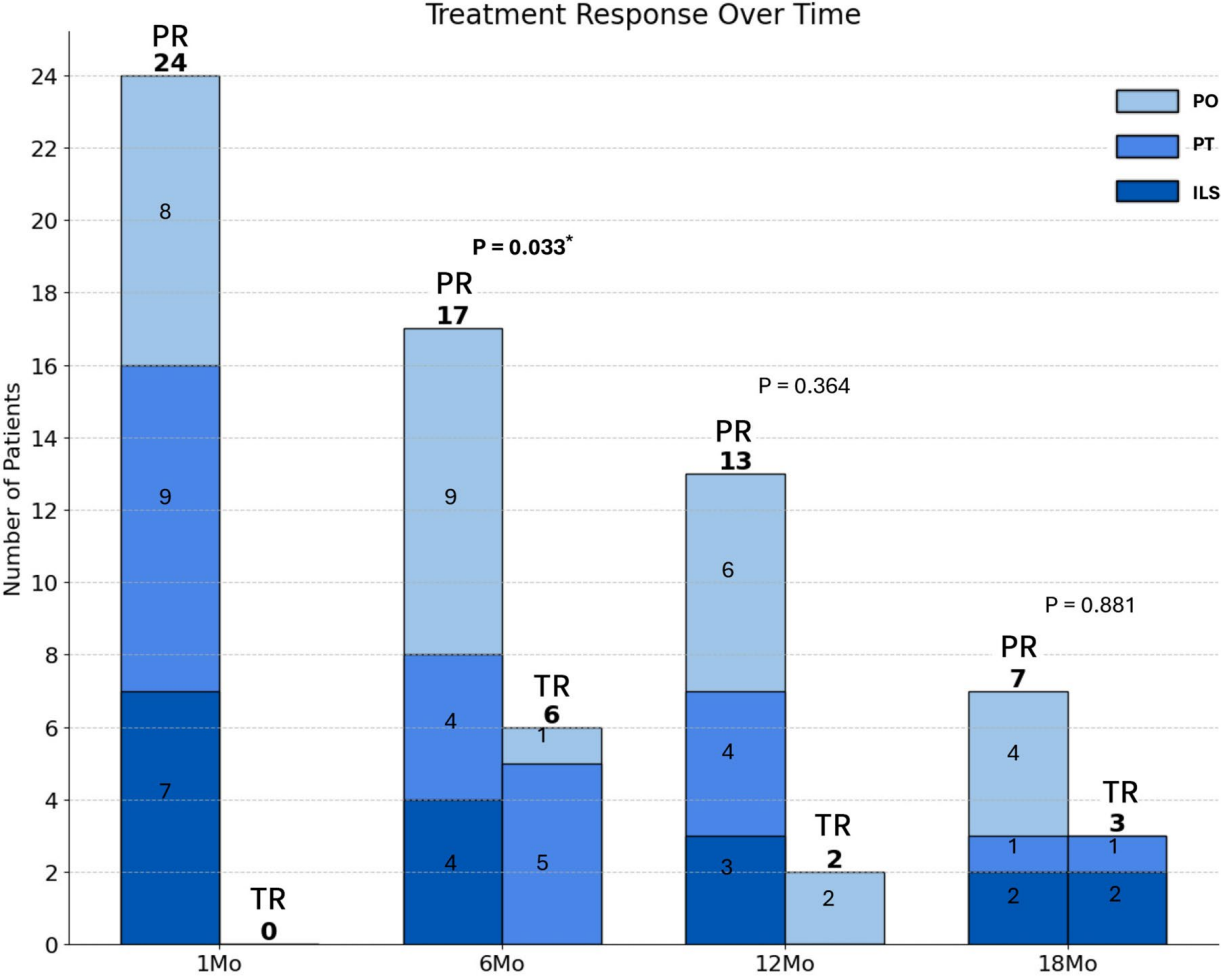
Treatment Response Over Time According to Treatment Modalities This stacked bar chart illustrates the treatment outcomes for MD patients at 1, 6, 12, and 18 months of follow-up. The bars represent the number of patients receiving different treatment modalities: PO (oral medications), PT (oral medication and topical treatments), and ILS (additional intralesional steroid injections). Treatment outcomes are categorized as PR (Poor Response) or TR (Treatment Response). At 1 month, all 24 patients showed PR across all treatment modalities. At 6 months, a symptom improvement was observed, with 6 patients showing TR. The majority of TR patients (5 out of 6) received a combination of oral and topical treatments compared to PR patients (*P* = 0.033). At 12 and 18 months, the number of patients with TR decreased slightly, and the differences in treatment modalities were not statistically significant (*p* = 0.364 and *p* = 0.881, respectively). The total number of patients decreased over time, due to monitoring cessation in TR cases or loss to follow-up. This figure demonstrates the potential benefits of combination therapy, particularly at the 6-month point

Combination therapy with oral and topical medications from the initial treatment phase was associated with better outcomes. At the 6-month evaluation, a statistically significant improvement in response rates was observed in the combined oral and topical treatment patients (*P* = 0.033), with five out of nine patients showing a positive response (Fig. 3).

### Generalized Linear mixed Model (GLMM)

The GLMM included age, sex, visit, triamcinolone injection, symptom duration, dyslipidemia, and the timing of first response as fixed effects, with patient-specific variability accounted for as a random effect to control for repeated measures within individuals.

The analysis revealed a significant negative association between the number of patient visits and clinical outcomes (*p* < 0.001), with more follow-up visits being associated with better outcomes. Dyslipidemia was significantly associated with worse outcomes (*p* = 0.006). Disease duration also showed a significant association with poorer outcomes (*p* = 0.020).

**Fig. 4:**
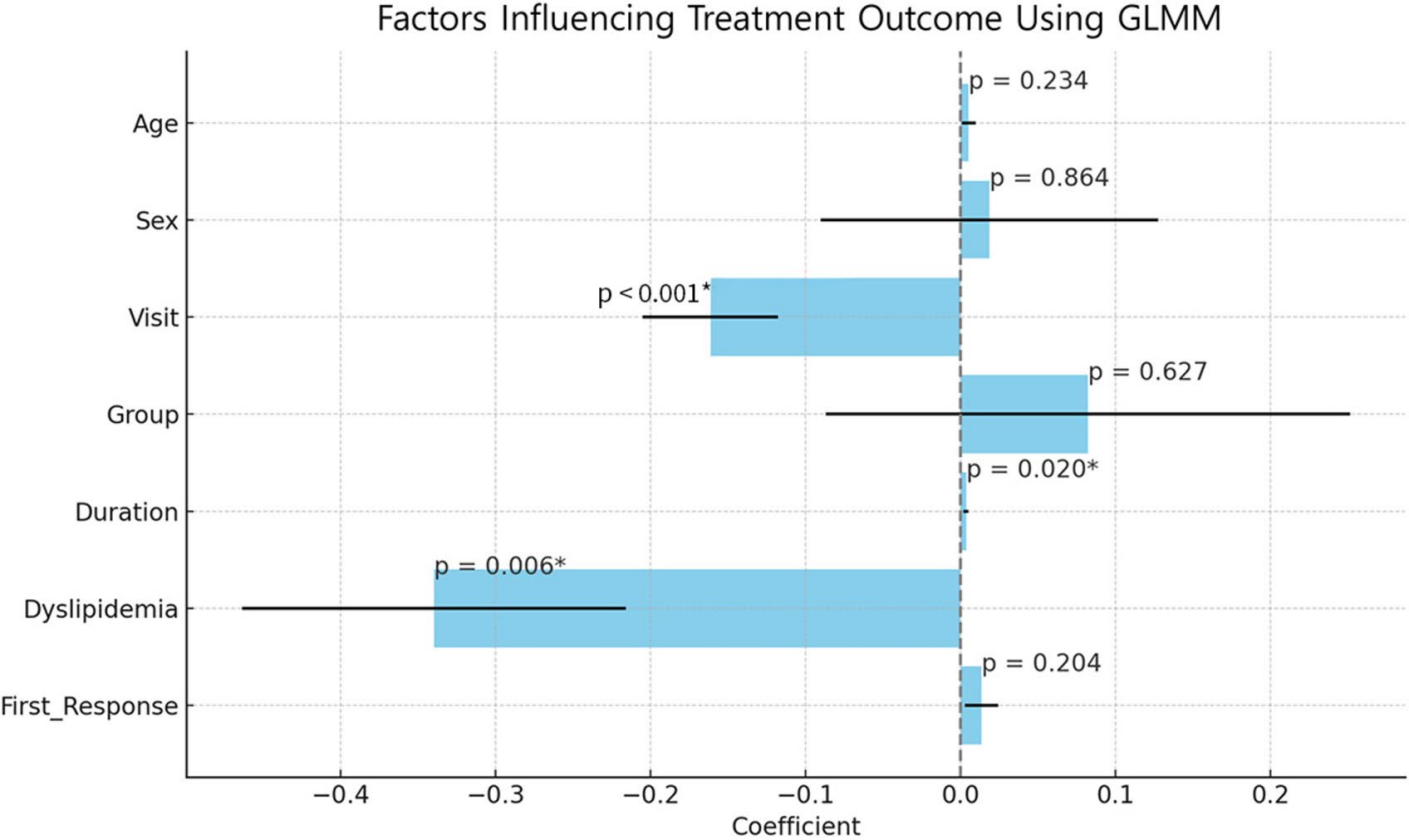
Factors Influencing Treatment Outcome Using GLMM This figure shows the results from the Generalized Linear Mixed Model (GLMM) analysis, examining factors influencing treatment outcomes in patients with MD. The x-axis represents the coefficient values for each variable, while the y-axis lists the variables included in the model: Age, Sex, Visit, Group, Duration, Dyslipidemia, and First Response. The horizontal bars represent the estimated coefficients for each factor, with error bars indicating the 95% confidence intervals. Statistically significant factors are marked with a p-value and an asterisk (*), denoting a p-value of less than 0.05. In this analysis, Visit, Duration, and Dyslipidemia were identified as significant predictors of treatment outcomes, with p-values of < 0.001, 0.020, and 0.006, respectively. This analysis showed that none of the medications, including minocin, isotretinoin, corticosteroid, tacrolimus ointment, ivermectin ointment, and ILS, had a statistically significant effect on treatment outcomes (*p* > 0.05 for all) Group, the grouping of patients based on the use of ILS; Visit, the number of outpatient visits; Duration, duration of symptoms; First response, the time period until the first treatment response

Age (*p* = 0.234), Sex (*p* = 0.864), triamcinolone injection (*p* = 0.627), and the time to first response (*p* = 0.204) were not significantly associated with clinical outcomes. The use of specific medications, including minocin, isotretinoin, triamcinolone injection, or protopic, ointment did not demonstrate a statistically significant impact on the outcomes. These findings are presented in Fig. 4, with significant factors marked by an asterisk (*) to indicate statistical significance at the 0.05 level.

The patient data, including clinical features and medical treatments in the TR group, are summarized in Table 4.

**Table 4:** Summary of patients with complete remission after treatment

Patients	Age (years)	Gender	Duration of Sx. (months)	Clinical features	Dyslipidemia	Treatment	Treatment time for first response (months)	Treatment time before complete response (months)
1	50s	Man	6	R UL: swelling, erythema, papules, and heating sensation	High LDL and High cholesterol level	Minocycline and tacrolimus for 1 month, partial response Minocycline, tacrolimus, and ivermectin for 6 months, partial response Isotretinoin and triamcinolone 2x, complete response	1	16
2	80s	Man	0.5	R UL: swelling and erythema	No	Prednisolone and tacrolimus for 1 month, no response Minocycline and tacrolimus for 3 months, complete response	1	4
3	60s	Man	3	R UL and LL: swelling, erythema, and heating sensation	No	Minocycline and tacrolimus for 1 month, partial response Isotretinoin and tacrolimus for 1 month, complete response	1	2
4	60s	Woman	6	L UL and LL: swelling and erythema	High LDL and High cholesterol level	Minocycline and tacrolimus for 1 month, partial response Minocycline and tacrolimus for 5 months, triamcinolone injection, complete response	3	6
5	50s	Woman	0.5	Bilateral: Facial swelling and erythema	No	Prednisolone and tacrolimus for 1 month, poor response Minocycline and tacrolimus for 3 months, complete response	1	4
6	60s	Man	1	L UL: swelling, erythema, and heating sensation	No	Prednisolone for 1 month, poor response Prednisolone and tacrolimus for 2 months, complete response	3	3
7	60s	Man	120	Bilateral UL: swelling and erythema	No	Minocycline, tacrolimus, and ivermectin for 1 month, poor response Minocycline, tacrolimus, and ivermectin for 11 months, triamcinolone injection 2x, partial response Isotretinoin for 6 months, complete response	3	14
8	60s	Man	3	R: Facial swelling and erythema	No	Minocycline and tacrolimus for 1 month, poor response Isotretinoin and tacrolimus for 5 months, triamcinolone injection, partial response Minocycline, tacrolimus, and ivermectin for 5 months, triamcinolone injection, complete response	3	17
9	60s	Man	3	R UL: swelling and erythema	N/A	Prednisolone for 1 month, poor response Minocycline and tacrolimus for 5 months, then ivermectin, triamcinolone injection, partial response Isotretinoin and tacrolimus for 3 months, triamcinolone injection, complete response	2	9
10	50s	Female	3	Bilateral UL and LL: swelling, erythema and papules	No	Isotretinoin and tacrolimus for 1 month, partial response Isotretinoin and tacrolimus for 3 months, complete response	1	4

*Sx* symptoms; *R* right; *L* left; *UL* upper lid; *LL* lower lid; *LDL* low-density lipid; *N/A* not available

## Discussion

This study provides novel insights into the clinical characteristics, prognostic factors, and treatment outcomes of MD in patients of South Korean descent, contributing to a deeper understanding of the disease within the Asian population. Our findings highlight the importance of early intervention, lipid management, and combination therapy in improving treatment outcomes for this challenging condition. Additionally, we were able to confirm the importance of monitoring the initial response after treatment.

One of the most significant findings of our study is the association between shorter symptom duration and better treatment outcomes. Patients who achieved TR had a significantly shorter mean duration of symptoms compared to those with PR. This suggests that early diagnosis and prompt initiation of treatment are crucial for managing MD effectively. The importance of early intervention in MD aligns with recent studies on chronic lymphedema and rosacea, which are considered underlying etiopathogenic factors of MD. For instance, Zhang et al. [20] demonstrated that prolonged exposure to LL-37, a peptide implicated in rosacea pathophysiology, results in irreversible dermal hyperplasia and persistent erythematous skin changes. Our findings support this concept, suggesting that early treatment of MD might prevent such irreversible changes, leading to more favorable outcomes [20, 21]. 

Another key finding of our study is the association between lower LDL-C levels and better treatment response. This novel observation suggests a potential link between lipid metabolism and MD pathophysiology or treatment response. The role of LDL in inflammatory skin conditions can be explained through its interaction with Toll-like receptors (TLRs), particularly TLR-2, which is elevated in rosacea-affected skin. LDL has been implicated in activating proinflammatory responses through TLR-2 signaling pathways. The association between high LDL levels and poor treatment response in our study suggests that the proinflammatory milieu enhanced by LDL might impede therapeutic efficacy in MD [22–25]. Recent meta-analyses have demonstrated a strong association between rosacea and cardiometabolic diseases, including dyslipidemia [26]. Our findings extend this concept to MD, suggesting that lipid management could be an important aspect of MD treatment.

Previous reports on MD treatment have highlighted that combination treatment and long-term management are crucial to ensure a positive response. Mireille et al. [21]. reported that a longer treatment duration of at least 6 − 12 months was associated with more satisfactory results. Combining eyelid reduction surgery with intralesional corticosteroids, isotretinoin with ketotifen, and tetracycline with systemic corticosteroids may help control symptoms [21]. In contrast, some studies have found that combination therapies show no correlation with treatment response [5, 12]. In this study, the treatment methods were divided into three groups: (1) Oral medications (isotretinoin, tetracycline, or corticosteroids) only; (2) oral medications combined with topical medications (tacrolimus or ivermectin); (3) treatment with additional ILS injections [7, 13, 18, 19, 27, 28]. In more than half of the patients who showed TR at the 6-month follow-up, combination treatment with oral and topical medications from the initial treatment and during the treatment period was a significant factor in achieving complete MD remission. Moreover, our study found that partial treatment response at 1 month was significantly associated with complete remission at the final visit. This observation underscores the value of early treatment response as a prognostic indicator, which has not been previously reported in MD literature.

Mireille et al. [21] also reported that 80% of patients treated with isotretinoin showed a positive response, highlighting its effectiveness in managing MD. However, it is noteworthy that many of these favorable outcomes are derived from case reports, where selection bias may play a role in highlighting responsive cases. In our study, which included a more diverse sample with an entirely Asian cohort, the response rate was around 30%. Additionally, our treatment protocol differed, incorporating adjunctive therapies such as topical treatments (Protopic, Ivermectin) and ILS injections—absent in previous protocols—which may have influenced outcomes. These factors—case severity, patient population, and treatment protocols—likely contributed to the differences in response rates observed between the studies.

The histopathological findings in our study, particularly the prevalence of perivascular or perifollicular lymphocytic infiltration, are consistent with previous reports [5, 11, 14, 28]. This was followed by dermal edema, focal granulomatous infiltrations, and telangiectasia. Notably, Demodex, the main microorganism that triggers the inflammatory reaction in rosacea, was found in one case of MD. Although this does not prove a definitive link between MD and Demodex, it is significant as it marks the first study where Demodex was identified in histological examinations of an Asian patient with MD. These findings illustrate the diverse histopathological spectrum of MD and its complex diagnosis and treatment.

Our observation of ANA positivity in 50% of patients, albeit at low levels, challenges conventional diagnostic criteria that associate ANA negativity with MD [4, 5, 28]. This finding is particularly intriguing when considering that ANA positivity in healthy individuals can reach up to 40% using indirect immunofluorescence techniques [29]. While this prevalence aligns with general population data, it suggests a need for a more nuanced approach to MD’s immunological profiling. Additionally, ANA is known to be present in over half of rosacea patients, which supports the idea that Morbihan disease may share a similar disease spectrum with rosacea [30]. This contrasts with previous studies that reported ANA negativity in MD, highlighting a potential area for reconsidering and updating the diagnostic criteria for MD. The presence of ANA in our study, without corresponding systemic or connective tissue diseases, underscores the potential for a more intricate immunological environment within MD than previously recognized.

The partial and variable responses to established treatments, such as tetracycline-based antibiotics, corticosteroids, and isotretinoin, present challenges for clinicians and patients. Our research indicates a trend towards improved outcomes with combined therapeutic approaches, potentially owing to a multifactorial impact on various pathogenic mechanisms in MD. The use of tacrolimus ointment, which acts in a manner similar to that of macrolide antibiotics, has emerged as a potentially effective component of combination therapy [27, 31]. Although not statistically significant, this finding suggests an anti-inflammatory effect worthy of further exploration.

It is crucial to acknowledge the limitations inherent in our study design. The retrospective nature and relatively small sample size may have introduced bias and limit the generalizability of our results. Additionally, the absence of a control group constrains our ability to definitively assess the long-term durability of treatment responses. Despite these limitations, our study offers valuable insights that have significant implications for both future research directions and clinical practice in MD management.

The identification of early symptom duration and LDL cholesterol levels as potential prognostic factors represents a notable advance in our understanding of MD. These findings not only provide tangible targets for clinical assessment but also raise intriguing questions about the underlying pathophysiology of the disease. The apparent link between lipid metabolism and MD outcomes suggests a previously unexplored avenue of research. Future studies investigating the relationship between lipid profiles, lymphatic dysfunction, and MD progression may reveal novel therapeutic targets and preventive strategies. Furthermore, the relationship between combination treatment regimens and early treatment response as factors for TR provides an important strategy for treatment approaches in patients with MD.

## Supplementary Information

Supplementary Material 1.

Supplementary Material 2.

## Data Availability

No datasets were generated or analysed during the current study.

## Abbreviations

MD: Morbihan Disease
TR: Treatment Response
PR: Poor Response
GLMM: Generalized Linear Mixed Model
REML: Restricted Maximum Likelihood
SPSS: Statistical Package for the Social Sciences
ROC: Receiver Operating Characteristic
AUC: Area Under the Curve
LDL-C: Low-Density Lipoprotein Cholesterol
ANA: Antinuclear Antibody
TLR: Toll-Like Receptor
LL-37: Cathelicidin Antimicrobial Peptide LL-37
