# Supplementary figures and images for: Novel prognostic factors and combination therapy outcomes in Morbihan disease: insights from an Asian population
Source: BMC Ophthalmol. 2024 Nov 13;24:496. doi: 10.1186/s12886-024-03758-2 (PMC11559205; doi:10.1186/s12886-024-03758-2)

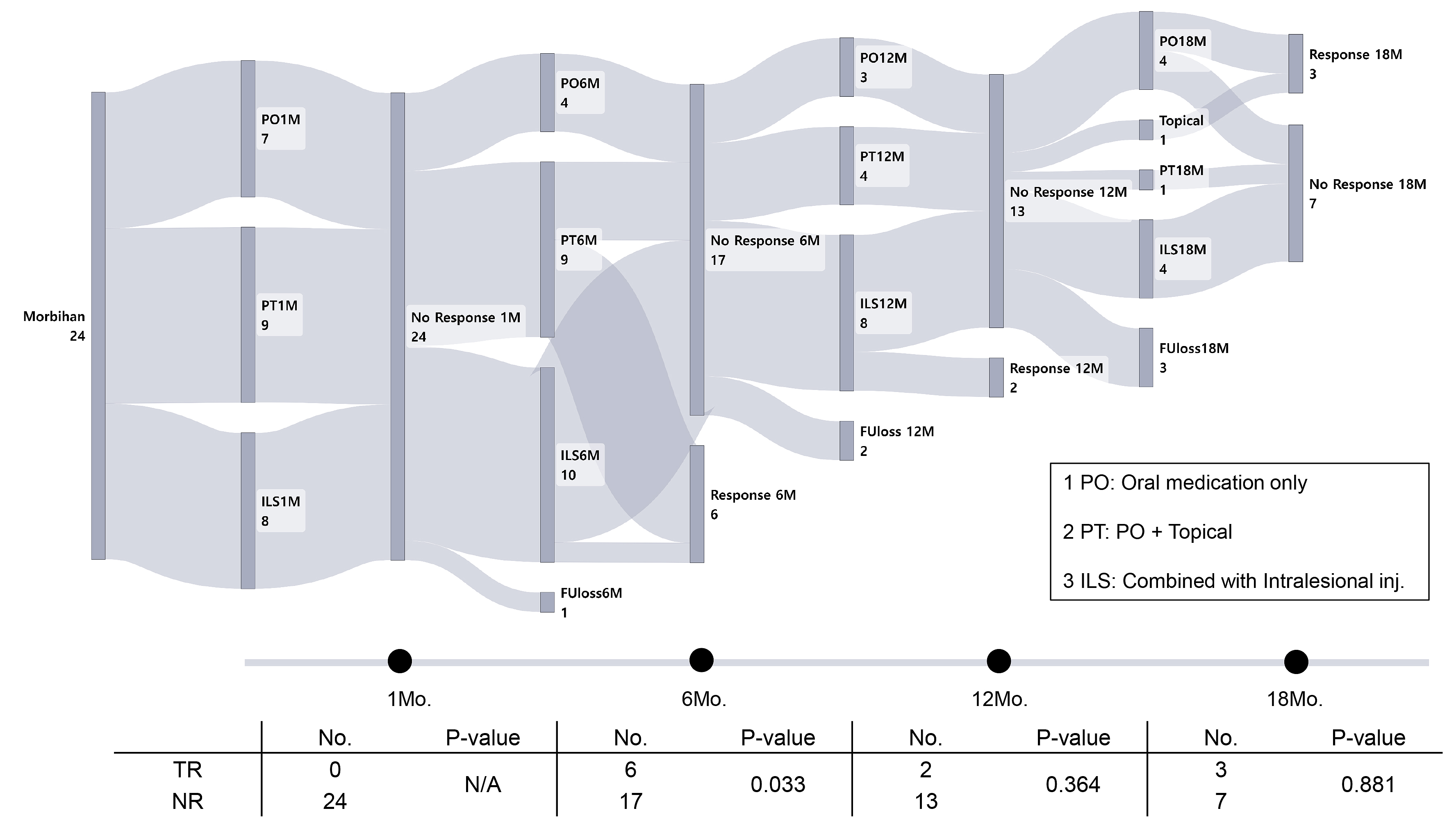

Supplement: Supplementary file 1 — Supplementary Material 1. [file 12886_2024_3758_MOESM1_ESM.jpg]
