# Supplementary material for: Novel prognostic factors and combination therapy outcomes in Morbihan disease: insights from an Asian population
Source: BMC Ophthalmol. 2024 Nov 13;24:496. doi: 10.1186/s12886-024-03758-2 (PMC11559205; doi:10.1186/s12886-024-03758-2)
Supplement: Supplementary file 2 — Supplementary Material 2. [file 12886_2024_3758_MOESM2_ESM.docx]

**Supplementary 1 file legends**

**Sankey diagram showing treatment progression for Morbihan disease based on medication.** Sankey diagram illustrates the treatment progression in patients with Morbihan disease according to the medications used. No patients showed a treatment response at 1 month. At 6 months, six patients exhibited a treatment response (TR), which was statistically significant and primarily observed in the combination treatment (PT group). By 12 months, an additional two patients, and by 18 months, three more patients showed TR, with no significant differences based on the medications used. FUloss, loss to follow-up; 1M, at 1-month; 6M, at 6-month; 12M, at 12-month; 18M, at 18-month; 1Mo., 1-month; 6Mo., 6-month; 12Mo., 12-month; 18Mo., 18-month
